# Increased Water-Soluble Yellow *Monascus* Pigment Productivity *via* Dual Mutagenesis and Submerged Repeated-Batch Fermentation of *Monascus purpureus*

**DOI:** 10.3389/fmicb.2022.914828

**Published:** 2022-06-09

**Authors:** Jie Bai, Zihan Gong, Meng Shu, Hui Zhao, Fanyu Ye, Chenglun Tang, Song Zhang, Bo Zhou, Dong Lu, Xiang Zhou, Qinlu Lin, Jun Liu

**Affiliations:** ^1^National Engineering Research Center of Rice and Byproduct Deep Processing, Central South University of Forestry and Technology, Changsha, China; ^2^Nanjing Sheng Ming Yuan Health Technology Co. Ltd., Nanjing, China; ^3^Jiangsu Institute of Industrial Biotechnology JITRI Co. Ltd., Nanjing, China; ^4^Biophysics Research Laboratory, Institute of Modern Physics, Chinese Academy of Sciences, Lanzhou, China; ^5^Hunan Provincial Key Laboratory of Food Safety Monitoring and Early Waring, Changsha, China

**Keywords:** yellow *Monascus* pigments, stability, mutation, response surface methodology, immobilized fermentation

## Abstract

*Monascus* pigments (MPs) have been used in the food industry for more than 2,000 years and are known for their safety, bold coloring, and physiological activity. MPs are mainly yellow (YMPs), orange (OMPs), and red (RMPs). In this study, a mutant strain *Monascus purpureus* H14 with high production of water-soluble YMPs (WSYMPs, *λ*_max_ at 370 nm) was generated instead of primary YMPs (*λ*_max_ at 420 nm), OMPs (*λ*_max_ at 470 nm), and RMPs (*λ*_max_ at 510 nm) produced by the parent strain *M. purpureus* LQ-6 through dual mutagenesis of atmospheric and room-temperature plasma and heavy ion beam irradiation (HIBI), producing 22.68 U/ml extracellular YMPs and 10.67 U/ml intracellular YMPs. WSYMP production was increased by 289.51% in optimal conditions after response surface methodology was applied in submerged fermentation. Application of combined immobilized fermentation and extractive fermentation improved productivity to 16.89 U/ml/day, 6.70 times greater than with conservative submerged fermentation. The produced WSYMPs exhibited good tone stability to environmental factors, but their pigment values were unstable to pH, light, and high concentrations of Ca^2+^, Zn^2+^, Fe^2+^, Cu^2+^, and Mg^2+^. Furtherly, the produced exYMPs were identified as two yellow monascus pigment components (monascusone B and C_21_H_27_NO_7_S) by UHPLC-ESI-MS. This strategy may be extended to industrial production of premium WSYMPs using *Monascus*.

## Introduction

*Monascus* pigments (MPs) are polyketide secondary metabolites produced by the genus *Monascus* and have been used as natural edible colorants in Asian food for thousands of years (Feng et al., 2012). Generally, MPs are red (RMPs, such as monascorubramine and rubropunctamine), orange (OMPs, such as rubropunctatin and monascorubrin), and yellow (YMPs, such as monascin and ankaflavin; Kim and Ku, 2018; Liu et al., 2019a). It has been reported that YMPs are an excellent colorants and are safe, with anti-inflammatory, anti-fatty, antitumor, and antioxidant functions (Wu et al., 2021; Huang et al., 2021b), improving alcoholic liver injury and preventing lipid accumulation (Zhou et al., 2019; Lai et al., 2021), with great prospects for use in food and pharmaceutical industries. However, YMPs are normally hydrophobic or alcohol-soluble intracellular pigments in submerged fermentation, increasing the cost of separation and purification, and limiting their application (Gong and Wu, 2016; Yang et al., 2020). Water-soluble yellow *Monascus* pigments (WSYMPs) and extracellular YMPs (exYMPs) have been studied extensively by researchers.

It has been reported that WSYMPs and exYMPs are not produced on a large scale as a result of their low yield and productivity (Qian et al., 2021; Huang et al., 2021b). Current research efforts often focus on increasing WSYMP/exYMP production through optimization of fermentation broth components, fermentation biotechnology, and chemical modification. Recently, extractive fermentation (EF) with the addition of nonionic surfactants in submerged fermentation of the *Monascus* genus has been widely used. The addition of Triton X-100 can change the mycelium morphology without harming cell growth in the EF of *M. anka* GIM 3.592, and increases exYMP production (Chen et al., 2017a,b). Xiong et al. (2015) intensified the transfer of intracellular YMPs into an extracellular nonionic surfactant micelle aqueous solution using EF and enhanced exYMP production. Huang et al. (2021b) increased WSYMP production by 69.68% compared to conventional fermentation by adding sodium starch octenyl succinate. In addition, high glucose stress can promote YMP biosynthesis and significantly increase extracellular YMP production (Huang et al., 2017c). Changing the extracellular oxidoreduction potential can also enhance WSYMP production in submerged fermentation of *Monascus ruber* CGMCC 10910 (Huang et al., 2017b). Chemical modification was performed by introducing H_2_SO_3_ into the double bond at the MP sidechain to produce WSYMPs with high stability over a wide pH range (Liu et al., 2020a). However, the application of these strategies, especially EF, to enhance exMPs has been problematic, increasing the cost of YMP purification.

Breeding of wild strains is most critical in microbial fermentation production of metabolites. Zhou et al. (2009) obtained a mutant strain with high YMP production through physical and chemical mutagenesis. Traditional methods of mutation breeding use chemical agents such as LiCl and nitrosoguanidine (Kodym and Afza, 2003; Sun et al., 2011). However, chemical mutagens can remain in the food industry and are harmful to human health; development of new and powerful mutagenesis methods is required. With the development of breeding biotechnology, atmospheric and room-temperature plasma (ARTP) mutagenesis systems have emerged as a novel methodology for strain breeding (Zhang et al., 2014). In our previous studies, mutant strains including *M. purpureus* M183 with high MP production, *M. purpureus* M630 with high exMP production, and *M. purpureus* M523 with high rice husk hydrolysate tolerance were generated using the ARTP screening system (Liu et al., 2019b, 2020b). In addition, heavy ion beam irradiation (HIBI), a novel and more efficient irradiation method for strain mutagenesis, has unique physical and biological advantages due to its high linear energy transfer and relative biological effectiveness, wide mutation spectrum, and high mutation rate (Gao et al., 2020; Guo et al., 2020; Zhang et al., 2022). Previous studies have demonstrated that HIBI produces a high mutation rate, mainly attributed to deletions, nucleotide exchange, and insertions (Chen et al., 2018). However, the combined application of ARTP and HIBI mutagenesis to enhance MP production has not been reported.

Generation of a mutant strain is difficult in industrial WSYMP production using *Monascus*. In this study, we obtained a mutant strain in WSYMP production through dual mutagenesis of HIBI (^12^C^6+^) and ARTP. Response surface methodology (RSM), EF, and immobilized fermentation (IF) were used to increase WSYMP yield and productivity.

## Materials and Methods

### Microorganisms and Chemical Materials

*Monascus purpureus* LQ-6 [CCTCC M 2018600, China Central for Type Culture Collection (CCTCC), Wuhan, China] was isolated from red mold rice obtained from a local market (Changsha, China) as the parent strain. The mutant strain *M. purpureus* H14 was obtained through dual mutagenesis of ARTP and HIBI. Analytical-grade chemicals were obtained from Sangon Biotech Co., Ltd. (Shanghai, China). Chemical reagents of vitamins were purchased from Coolaber (Beijing, China). Potatoes used to produce potato dextrose agar (PDA) medium were purchased from a local market (Changsha, China).

### Mutagenesis and Screening

*Monascus purpureus* strains were cultured on PDA medium (200 g/L potato, 20 g/L glucose, and 20 g/L agar) for 7 days at 30°C in darkness. The mycelium on a PDA Petri dish containing a fully grown culture of *M. purpureus* strain was aseptically scraped and inoculated in a 100-ml conical flask containing 20 ml of sterile water and glass beads, and agitated for 3 min to prepare the spore suspension; the concentration was adjusted to approximately 10^7^ spores/ml.

Heavy ion beam irradiation was performed using carbon ion beams (^12^C^6+^, 80 MeV/u, and 20 keV/μm) at the Heavy Ion Research Facility in Lanzhou (HIRFL), Institute of Modern Physics, Chinese Academy of Sciences. The spore suspension (1.5 ml) was transferred into a 35-mm irradiation dish. The detailed steps were reported by Gao et al. The irradiation dose was set as 0, 50, 100, 150, 200, 250, and 300 Gy (Gao et al., 2020). The ARTP mutagenesis process was presented in our previous study (Liu et al., 2019b). The conditions were the same as in our previous study, except the variable parameter treatment time *T* was set to 0, 40, 80, 120, 160, 200, 240, and 280 s. After mutagenesis, 100 μl of the spore suspension was spread onto PDA plates and incubated at 30°C in darkness to generate the mutant strains. The fatality and survival rates were analyzed by normalizing the colony counts of irradiated spores on PDA plates with those of untreated spores.

The spore suspension (5 ml, 10^7^ spores/ml) was inoculated in a 250-ml conical flask containing 45 ml of screening fermentation broth (80 g/L rice flour, 2.5 g/L yeast extract, 2.5 g/L peptone, 5 g/L KH_2_PO_4_, and 0.01 g/L FeSO_4_•7H_2_O), incubated for 10 days at 30°C, and agitated at 150 rpm in the dark.

### Fermentation Biotechnologies

Conventional submerged batch fermentation (SBF) was conducted as follows: 5 ml of the spore suspension (10^7^ spores/ml) was inoculated in a 250-ml conical flask containing 45 ml of fermentation broth (50 g/L glucose, 2 g/L malt extract, 10 g/L peptone, 2.5 g/L KH_2_PO_4_, 5 g/L NaNO_3_, 1 g/L MgSO_4_•7H_2_O, 2 g/L ZnSO_4_•7H_2_O, and pH 3.8), incubated for 10 days at 30°C, and agitated at 150 rpm in the dark.

Recent studies (Hu et al., 2012; Chen et al., 2017a; Lu et al., 2021) have revealed that the concentration of 40 g/L of Triton X-100 is the preferred effective strategy for improving MP production in extractive SBF by parent strain. Thus, Triton X-100 (40 g/L) was added to the optimized conventional submerged batch-fermentation broth (20 g/L glucose, 12.29 g/L malt extract, 15 g/L peptone, 2.5 g/L KH_2_PO_4_, 5 g/L NaNO_3_, 0.2 g/L MgSO_4_•7H_2_O, 0.32 g/L ZnSO_4_•7H_2_O, 0.1 g/L MnSO_4_•H_2_O, 0.15 g/L CaCl_2_, 0.15 g/L FeSO_4_•7H_2_O, 0.4 g/L vitamin B5, and pH 4.8) using RSM to construct the extractive SBF.

Immobilized fermentation was performed according to the method in our previous study, with several modifications (Liu et al., 2019b; Zhang et al., 2022). The spores of mutant strain *M. purpureus* H14 were immobilized in different ratios of sodium alginate (SA) and polyvinyl alcohol (PVA) solution [3% (wt/wt):0% (wt/wt), 2.5% (wt/wt):0.5% (wt/wt), 2% (wt/wt):1% (wt/wt), and 1.5% (wt/wt):1.5% (wt/wt)] instead of in 3% (wt/wt) sodium alginate solution.

The kinetics of SBF and repeated-batch fermentation (RBF) of *M. purpureus* H14 were determined in a 1,000-ml conical flask containing 300 ml of optimized fermentation broth. During fermentation, 10-ml samples were collected to measure metabolite concentrations.

### Experimental Design and Statistical Analysis

Response surface methodology was used to optimize four conventional SBF broth components (ZnSO_4_•7H_2_O, MnSO_4_•H_2_O, malt extract, and vitamin B5) to enhance WSYMP production after fermentation of *M. purpureus* H14. The Box–Behnken design (BBD) was used to optimize the components. The four independent variables used in the experimental design were ZnSO_4_•7H_2_O (0.1–0.5 g/L), MnSO_4_•H_2_O (0.05–0.2 g/L), malt extract (5–15 g/L), and vitamin B5 (0.3–0.5 g/L); three levels were used to optimize the fermentation process.

### Physicochemical Property Assays

To determine the polarity of YMPs produced by the mutant strain *M. purpureus* H14, 1 ml of fermentation medium was collected and centrifuged at 10,000 rpm for 5 min, followed by the addition of 1 ml of pure water or ethyl acetate to the fermentation medium and ankaflavin-methanol solution, respectively.

As in our previous study, the YMP alcohol solution was generated using the adsorption–separation system with the macroporous adsorption resin LX300C (Liu et al., 2019b). Resin LX300C (2 g) was added to a 100-ml conical flask containing 50 ml of the centrifuged fermentation medium to adsorb the YMPs at 30°C for 24 h and agitated at 150 rpm in the dark. The YMPs were desorbed using 70% (v/v) alcohol eluent in the same conditions. The same volume of pure water was added to the YMPs prepared by freeze-drying to generate the WSYMP solution.

The pH of the WSYMP solution and ankaflavin-methanol solution (the concentration was adjusted to 1 g/L, equal to 30.08 U/ml) was regulated from 1 to 14 with 1 mol/L HCl or NaOH. A full-band scan was performed to determine the maximum absorption wavelength and measure the WSYMPs with different concentrations (1–100 mmol/L) of metal ions added to the WSYMP solution, including Na^+^, Mg^2+^, K^+^, Ca^2+^, Zn^2+^, Cu^2+^, and Fe^2+^. Five milliliters of WSYMP solution and ankaflavin-methanol solution was poured into a 60 mm × 20 mm culture dish before irradiation (3,000 lx of light intensity, 30 cm of irradiation distance) in a constant-temperature incubator (adjusted to 30°C) with incandescent LED lamps; the group left in darkness (0 lx) was used as the control. WSYMP solution (5 ml) was treated in an electro-thermostatic water bath at different temperatures (25°C–90°C) to evaluate the pH, metal ions, temperature, and visible light stability.

### Composition of YMPs

Yellow *Monascus* pigments were detected by ultra-high-performance liquid chromatography (Dionex Ultimate 3000 UHPLC). The mobile phase was 0.1% formic acid (20%) and methanol (80%) at a flow rate of 0.5 ml/min, with separation carried out using an Eclipse Plus C18 column (100 mm × 4.6 mm, 3.5 μm) at 30°C, run time for 15 min and 5 μl of injection volume. Mass spectrometry was carried out by Ultimate 3000 UHPLC-Q Exactive (Thermo Scientific, United States) equipped with an HESI source. The parameters were set as follows: 10 ml/min of aux gas flow rate, 40 ml/min of sheath gas flow rate, capillary temperature at 300°C, 3.8 kv of capillary voltage, and 50% S-Lens. Each sample was analyzed in positive modes with a mass scan range of 100–800 m/z, and resolution was set at 70,000.

### Determination of Metabolites and Calculations

The concentrations of glucose, biomass, and YMPs were measured according to our previously described methods (Liu et al., 2019a,b). The residual glucose concentration was determined using the standard 3, 5-dinitrosalicylic acid method. The YMP concentration was analyzed by measuring the absorbance of the supernatant at 370 nm, which included intracellular YMP content (inYMPs) and exMP content.

For the data analysis, *F* test was applied to evaluate the effect of independent variables on the RSM; the significant results were identified by a value of *p* < 0.05; the fitness of second-order model was evaluated by multiple correlation coefficient (*R*^2^) and adjusted *R*^2^(*R*^2^adj.). Each experiment was repeated at least three times; the numerical data are presented as the mean ± SD.

## Results and Discussion

### Obtaining Mutant Strain

**Figure 1:**
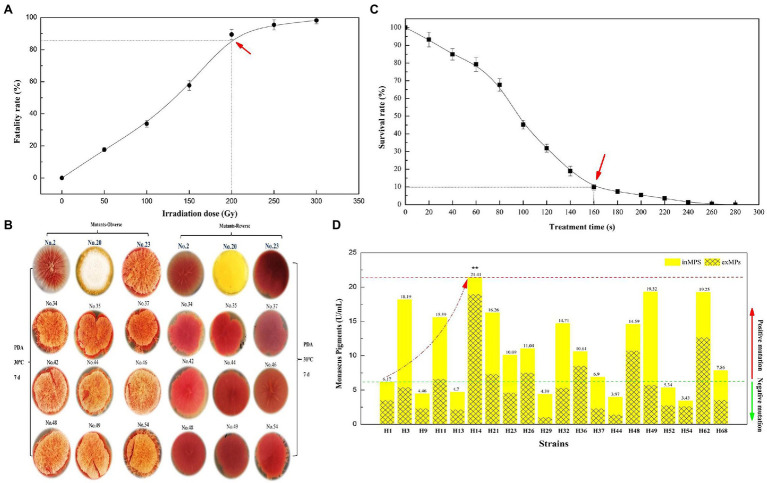
Fatality rate **(A)** and survival rate **(C)** of parent strain *Monascus purpureus* LQ-6 subjected to heavy ion beam irradiation (HIBI) and atmospheric and room-temperature plasma (ARTP) mutation breeding system for various irradiation dose and treatment times, respectively; the morphology and color of mutant strains on potato dextrose agar (PDA) plates **(B)**; the production of total yellow *monascus* pigments by the mutant strains obtained *via* using ARTP by submerged batch fermentation (SBF) for 10 days at 30°C and agitated at 150 rpm in the dark **(D)**.

From Figure 1A, the fatality rate of parent strain *M. purpureus* LQ-6 was 89.64% (approximately 90%) at an irradiation dose of 200 Gy. Over 250 Gy, the fatality rate exceeded 95.47%; at 300 Gy, the active *M. purpureus* LQ-6 spores were almost completely killed. According to modern theory of breeding andpositive/negative mutation rate, a 90% fatality rate was set as the target (Dong et al., 2017). Thus, an irradiation dose of 200 Gy was chosen for further evaluation. The morphology and color of the mutant strains were observed for 7 days during growth on PDA plates. We found three distinct mutant strains among hundreds of potential mutant colonies; mutant strain No. 2 exhibited a smooth surface (almost no hypha), and the color was light in comparison with others. From the reversed colony on the PDA plate, mutant strain No. 23 exhibited the reddest color, and the hypha was abundant. Mutant strain No. 20 exhibited niveous hypha, but the PDA agar broth was yellow (Figure 1B). It remained yellow after inoculation with multiple passages of mutant strain No. 20; thus, this mutant strain (denoted as H1) was selected for further YMP production by ARTP.

From the survival rate curve for mutant strain H1 by ARTP, the mortality rate reached 90.28% at *T* = 160 s (Figure 1C), which was different from reported (*T* = 180 s) in our previous study (Liu et al., 2019b), illustrating that the sensitivity of different strains to ARTP mutagenesis is distinct. Subsequently, 68 mutant strains were selected for the determination of the target mutant strain *via* SBF after preliminary investigation of the morphology and color of mutant strains generated by ARTP mutagenesis with T = 160 s. Figure 1D indicates that the mutant strain *M. purpureus* H14 produced the highest concentration of YMPs (*λ*_max_ at 370 nm, 21.41 U/ml) by SBF in the screening fermentation broth, which was approximately 3.47 times greater than the concentration produced by the mutant strain *M. purpureus* H1. However, the primary YMPs (*λ*_max_ at 420 nm), OMPs (*λ*_max_ at 470 nm), and RMPs (*λ*_max_ at 510 nm) were produced by the parent strain *M. purpureus* LQ-6 in these conditions. The ratio of exYMPs to inYMPs was 7.81, indicating that mutant strain *M. purpureus* H14 with high production of exYMPs was obtained through dual mutagenesis of HIBI and ARTP.

### Effect of Glucose Concentration and pH on YMP Production

It is known that fermentation parameters, including substrate concentration and pH, play a vital role in targeted product biosynthesis. Pigment production by the genus *Monascus* has been reported to be significantly affected by glucose concentration (Chen and Johns, 1994; Wang et al., 2017a). The composition and color characteristics of *Monascus* pigments can be controlled by pH and nitrogen sources in SBF (Shi et al., 2015). Based on these findings, we determined the optimal original glucose concentration and pH of the fermentation broth for *M. purpureus* H14 in SBF; the initial glucose concentration was increased from 10 to 70 g/L (the raw concentration was 50 g/L), and the pH of the conventional SBF broth was adjusted from 2.8 to 6.8 (the raw pH was 3.8).

**Figure 2:**
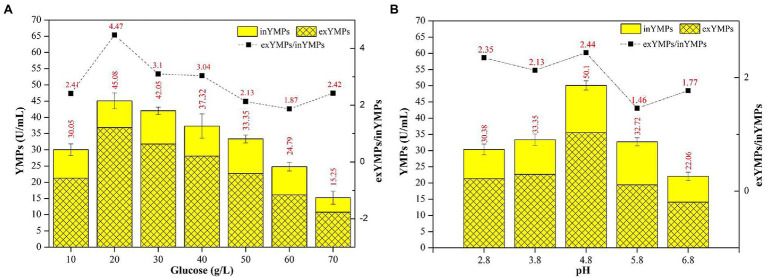
The effect of initial glucose concentration **(A)** and pH **(B)** in the fermentation broth on the production of yellow *monascus* pigments (YMPs) and the ratio of extracellular YMPs to intracellular YMPs.

Figure 2 shows the effect of initial glucose concentration and pH on YMP production *via* SBF. The total YMP production and the ratio of exYMPs to inYMPs (exYMPs/inYMPs) in SBF for *M. purpureus* H14 were significantly affected by regulating the original glucose concentration and pH. YMP production and the exYMPs/inYMPs ratio were increased by 35.17 and 109.86% (to 45.08 U/ml and 4.47), respectively, when the initial glucose concentration was 20 g/L, compared to a raw concentration of 50 g/L (33.35 U/ml and 2.13). Figure 2A also shows that YMP production decreased as the glucose concentration increased beyond 30 g/L. When the glucose concentration was 70 g/L, total YMP production decreased to 15.25 U/ml, but a high exYMPs/inYMPs ratio was maintained. Figure 2B shows that YMP production and the exYMPs/inYMPs ratio increased by 50.22 and 14.55% (to 50.10 U/ml and 2.44), respectively, when the initial pH was adjusted to 4.8, compared with a raw pH of 3.8 (33.35 U/ml and 2.13). The exYMPs/inYMPs ratio decreased when the initial pH was greater than 5.8; the change was insignificant when the pH was less than 4.8. It has been reported that glucose (and pH) can control the oxidoreduction potential (ORP) level, which can further regulate the ratio of NADH/NAD^+^ and intracellular enzyme activity (Wang et al., 2017b). High glucose stress can promote YMP biosynthesis by changing ORP levels (Wang et al., 2017b). High exYMP production was generated by adding 30 g/L glucose in fed-batch fermentation (Hu et al., 2012). Chen and Johns reported that the growth of *M. purpureus* and the biosynthesis of ankaflavin (a type of YMP) were favored at low pH (4.0); the growth and biosynthesis of other pigments were independent of pH (Chen and Johns, 1993).

The optimal initial glucose concentration and pH in SBF broth were determined to be 20 g/L and 4.8, respectively, for maximum exYMP yield and cost-efficiency for industrialized application.

### Optimization of Fermentation Medium by RSM

It is known that RSM can effectively increase the yield of a target product using liquid-state fermentation (or submerged fermentation). YMP production in this study using *M. purpureus* H14 with different concentrations of vitamins, nitrogen, and metal ions is shown in Table 1. According to the experimental data, vitamin B5 (0.4 g/L), malt extract (10 g/L), ZnSO_4_•7H_2_O (0.2 g/L), and MnSO_4_•H_2_O (0.1 g/L) significantly upregulated YMP production (increased by 90.64, 185.31, 153.61, and 123.42%, respectively). In order of importance to YMP production, these can be ranked as malt extract (a representative nitrogen source), ZnSO_4_•7H_2_O, MnSO_4_•H_2_O (representative of metal ions), and vitamin B5 (representative of growth cofactors). In addition, we found that the ratio of exYMPs to total YMPs was generally approximately 70%, which was equal to the ratio of exYMPs/inYMPs at 2.33 (similar to that with different initial pH). From the glucose and pH regulation findings, we confirmed that *M. purpureus* H14 was a high-production exYMP mutant strain, possibly due to the high cell permeability or the hydrophilia of the YMPs (WSYMPs). Thus, an increase in YMP production by RSM was imminent.

**Table 1:** The effect of different fermentation medium compounds on the production of yellow *Monascus* pigments in submerged fermentation for 10 days at 30°C and agitated at 150 rpm in the dark.

Composition	Compound	Concentration (g/L)	inMPs (U/ml)	exMPs (U/ml)	T-MPs (U/ml)	exMPs/T-MPs (%)	Increased by (%)
CK	–	–	10.67	22.68	33.35	68.01	–
Growth factor	B1	0.2	17.79	29.82	47.61	62.63	42.76
	B3	0.2	15.06	27.86	42.52	65.52	27.50
	B5	0.2	15.10	36.53	51.63	70.75	54.81
	B6	0.2	12.04	28.22	40.26	70.09	20.72
	VC	0.2	14.57	32.28	46.85	68.90	40.48
	B5	0.1	10.76	24.84	35.60	69.78	6.75
		0.2	15.10	36.53	51.63	70.75	54.81
		0.3	15.28	40.17	55.45	72.45	66.27
		0.4	19.09	44.49	63.58	69.98	90.64
		0.5	15.38	33.24	48.62	68.37	45.79
Nitrogen source	Peptone	5	23.66	36.70	60.36	60.80	80.99
		10	26.80	46.50	73.30	63.44	119.79
		15	36.76	52.80	89.56	58.95	168.55
		20	21.48	46.35	67.83	68.33	103.39
	Malt extract	5	27.64	54.65	82.29	66.41	146.75
		10	35.80	59.35	95.15	62.37	185.31
		15	26.46	48.45	74.91	64.68	124.62
		20	23.84	44.55	68.39	65.14	105.07
	NaNO3	1	14.45	28.20	42.65	51.24	27.89
		3	19.90	45.65	65.55	69.64	96.55
		5	28.40	51.50	79.90	64.46	139.58
		7	18.59	39.46	58.05	67.96	74.06
		9	11.85	32.80	44.65	73.46	33.88
Metal ion	Mg^2+^	0.05	15.62	29.30	44.92	65.23	34.69
		0.1	15.58	36.73	52.31	70.22	56.85
		0.2	17.65	43.27	60.92	71.03	82.67
		0.5	19.59	36.81	56.40	65.26	69.12
	Mn^2+^	0.01	13.65	39.12	52.77	74.13	58.23
		0.05	15.17	48.16	63.33	76.05	89.90
		0.1	19.63	54.88	74.51	73.65	123.42
		0.2	17.46	34.08	51.54	66.12	54.54
	Fe^2+^	0.5	12.35	29.57	41.92	70.55	25.70
		1	13.20	34.15	47.35	72.13	41.98
		1.5	13.08	39.76	52.84	75.25	58.44
		2	16.32	28.04	44.36	63.22	33.01
	Ca^2+^	0.5	9.73	24.23	33.96	71.35	1.83
		1	13.01	30.91	43.92	70.38	31.69
		1.5	17.16	36.92	54.08	68.26	62.16
		2	11.60	25.30	36.90	68.56	10.64
	Zn^2+^	0.05	17.99	47.03	65.02	72.33	94.96
		0.1	22.41	52.65	75.06	70.15	125.07
		0.2	26.75	57.83	84.58	68.37	153.61
		0.5	18.78	42.22	61.00	69.22	82.91

The data were expressed as the mean from three experiments.

Recently, the use of RSM to increase MP production using *Monascus* spp. has increased. Our previous study showed that the total MPs and exMPs were increased by 33.38 and 150.32%, respectively, *via* SBF of *M. purpureus* (Liu et al., 2019b). Srivastav et al. (2015) used RSM to optimize a sweet potato-based medium to increase red MP production using *M. purpureus*. The four medium compounds, vitamin B5, malt extract, ZnSO_4_•7H_2_O, and MnSO_4_•H_2_O, were selected for RSM.

**Figure 3:**
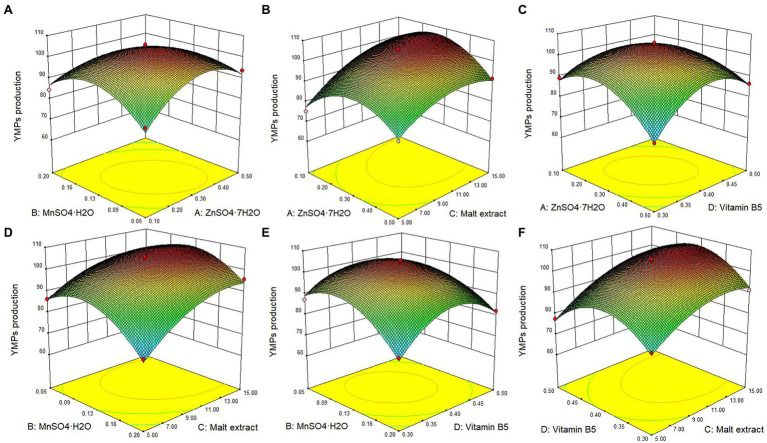
Response surface plot of the effect of the interaction of ZnSO4•7H2O, MnSO4•H2O, malt extract, and vitamin B5 on the YMPs production in submerged fermentation by mutant strain *M. purpureus* H14. Effect of ZnSO4•7H2O and MnSO4•H2O **(A)**, effect of ZnSO4•7H2O and malt extract **(B)**, effect of ZnSO4•7H2O and vitamin B5 **(C)**, effect of MnSO4•H2O and malt extract **(D)**, effect of MnSO4•H2O and vitamin B5 **(E)**, and effect of malt extract and vitamin B5 **(F)**.

According to the BBD for these four compounds with a three-level optimization (−1, 0, and 1), 29 experimental runs were conducted in this study (Table 2), and the experimental YMPs production were marked with arrows in Figure 3. State-ease Design Expert 8.0.6 software was used to analyze the relationship between YMP production (*Y*) and parameters (*X*_i_) in each experimental run; the following multiple nonlinear regression model (Equation 1) was generated to express the relationship.


(1) $$\begin{array}{l} Y=104.69-1.18X_{1}-3.80X_{2}\\ \;+7.88X_{3}-0.067X_{4}-6.56X_{1}X_{2}-2.80X_{1}\;X_{3}\\ \;+5.91X_{1}X_{4}+2.62X_{2}X_{3}+3.59X_{2}X_{4}\\ \;+1.11X_{3}X_{4}-11.30{X_{1}}^{2}-10.68{X_{2}}^{2}\\ \;-6.48{X_{3}}^{2}-12.39{X_{4}}^{2} \end{array}$$

**Table 2:** Box–Behnken design for process parameters of yellow *monascus* pigments production by *M. purpureus* H14 in submerged fermentation for 10 days.

Compounds	Symbol	Coded levels				
		−1		0	1	
ZnSO_4_•7H_2_O	A	0.1		0.3	0.5	
MnSO_4_•H_2_O	B	0.05		0.13	0.2	
Malt extract	C	5		10	15	
Vitamin B5	D	0.3		0.4	0.5	
Run order	A	B	C	D	YMPs (U/ml)	
					Experimental	Predicted
1	0	1	0	1	82.06	81.34
2	−1	0	1	0	98.56	98.76
3	−1	0	0	1	77.92	76.20
4	0	0	0	0	103.28	104.69
5	0	0	−1	1	77.52	76.76
6	−1	0	−1	0	75.26	77.41
7	1	0	1	0	91.66	90.82
8	0	0	0	0	105.88	104.69
9	0	−1	1	0	97.18	96.90
10	1	−1	0	0	93.67	91.89
11	0	1	−1	0	74.38	73.24
12	0	0	1	−1	91.48	92.65
13	0	1	1	0	95.71	94.23
14	0	−1	0	−1	75.37	74.30
15	0	−1	0	1	79.38	81.77
16	−1	−1	0	−1	82.94	81.13
17	1	0	0	−1	73.98	73.98
`18	0	0	0	0	104.56	104.69
19	1	1	0	0	68.94	71.17
20	−1	0	0	−1	89.16	88.15
21	0	−1	0	−1	87.04	89.07
22	0	0	−1	−1	80.24	79.12
23	1	0	−1	0	79.54	80.65
24	0	0	0	0	104.37	104.69
25	−1	1	0	0	84.45	86.64
26	1	0	0	1	86.38	85.66
27	0	−1	−1	0	86.32	86.07
28	0	0	0	0	105.36	104.69
29	0	0	1	1	93.21	94.75

The data were expressed as the mean from three experiments.

where *Y* represents the response values of YMP production, and *X*_1_, *X*_2_, *X*_3_, and *X*_4_ represent ZnSO_4_•7H_2_O, MnSO_4_•H_2_O, malt extract, and vitamin B5, respectively.

The statistical significance of the regression model equation was evaluated by the *F* test analysis of variance; the associated *p*-values were lower than 0.001 for YMPs production, indicating that the developed model and the terms were statistically significant; besides, the value of lack of fit was 0.0704, higher than 0.05, demonstrated the precision and the accuracy of the constructed models; what is more, the data show that MnSO_4_•H_2_O and malt extract were the principal factors on YMPs production (Table 3). Based on the experimental data, there were six response surface graphs in multiple nonlinear quadratic regression models. The adequacy and accuracy of the generated model were evaluated using coefficient of determination (*R*^2^), adjusted *R*^2^ (*R*^2^_adj_), predicted *R*^2^ (*R*^2^_pred_), and coefficient of variation (CV%). In this study, the results of ANOVA for RSM quadratic model showed that the value of *R*^2^ was 0.9842 for YMPs production and *R*^2^_adj_ was 0.9683, which was high and significantly close to the *R*^2^ value. These results indicated that the predicted model was appropriate. In addition, the value of *R*^2^_pred_ (0.9138 for YMPs production) was in reasonable agreement with that of *R*^2^_adj_, which also demonstrated that the predicted values of YMP productions were accurate. Furthermore, adequate precision (Adeq. Pre.) of 24.113 indicated an adequate signal (greater than 4 is desirable); this model can be used to navigate the design space. Thus, from Figure 3, the YMPs production point prediction was used in the Design Expert software to determine the four medium parameters with optimized values. The optimal values of ZnSO_4_•7H_2_O, MnSO_4_•H_2_O, malt extract, and vitamin B5 were determined as 0.32, 0.1, 12.29, and 0.4 g/L, respectively. In optimized fermentation conditions for 10 days, triplicate experiments resulted in a mean YMP production of 104.67 U/ml, which was 98.26% of the predicted value. YMP production and exYMP production increased by 213.85 and 289.51%, respectively, using RSM, and the ratio of exYMPs/inYMPs increased from 2.13 to 5.41. These results indicate that RSM dramatically increased exYMP production; the yield and productivity of exYMPs were 4,417 U/g (glucose) and 8.83 U/ml/day. Subsequently, IF and EF were performed together to increase exYMP productivity.

**Table 3:** ANOVA for the effect of ZnSO_4_•7H_2_O, MnSO_4_•H_2_O, vitamin B5, and malt extract on yellow *monascus* pigment production and regression coefficients.

Source	Sum of squares-type III	df	Mean square	*F* value	*p* value Prob > *F*	Significance
Model	3249.06	14	232.08	62.12	<0.0001	**
A (ZnSO_4_•7H_2_O)	16.61	1	16.61	4.45	0.0535	–
B(MnSO_4_•H_2_O)	173.43	1	173.43	46.42	<0.0001	**
C (malt extract)	744.82	1	744.82	199.36	<0.0001	**
D (vitamin B5)	0.053	1	0.053	0.014	0.9066	–
AB	172.13	1	172.13	46.07	<0.0001	**
AC	31.25	1	31.25	8.36	0.0118	*
AD	139.71	1	139.71	37.40	<0.0001	**
BC	27.41	1	27.41	7.34	0.0170	*
BD	51.48	1	51.48	13.78	0.0023	*
CD	4.95	1	4.95	1.33	0.2690	–
*A* ^2^	828.56	1	828.56	221.77	<0.0001	**
*B* ^2^	739.81	1	739.81	198.02	<0.0001	**
*C* ^2^	272.13	1	272.13	72.84	<0.0001	**
*D* ^2^	996.09	1	996.09	266.61	<0.0001	**
Residual	52.31	14	3.74			
Lack of fit	48.33	10	4.83	4.87	0.0704	–
Pure error	3.97	4	0.99			
Corrected total	3301.36	28				
*SD*	1.93					
Mean	87.79					
CV%	2.20					
*R* ^2^	0.9842					
*R* ^2^_adj_	0.9683					
*R* ^2^_pred_	0.9138					
Adep. pre	24.113					

CV, coefficient of variation; *R*^2^, coefficient of determination; *R*^2^_adj_, adjusted R^2^; *R*^2^_pred_, predicted *R*^2^; and Adep. Pre, adequate precision.

** p < 0.01 highly significant.

* p < 0.05, significant; −, not significant.

### Improvement of exYMP Productivity *via* RBF

Extractive fermentation has been proven to efficiently increase the production of *Monascus* pigments in submerged fermentation of *Monascus* spp.; the exMPs increased by 127.48% with the addition of 3 g/L Triton X-100 in submerged fermentation of *M. purpureus* DK (Lu et al., 2021). Our previous study prevented further improvement of the yield and productivity of exMPs by IF (Liu et al., 2019b). In this study, a novel RBF biotechnology was developed by combining EF and IF to significantly increase exYMP production.

**Figure 4:**
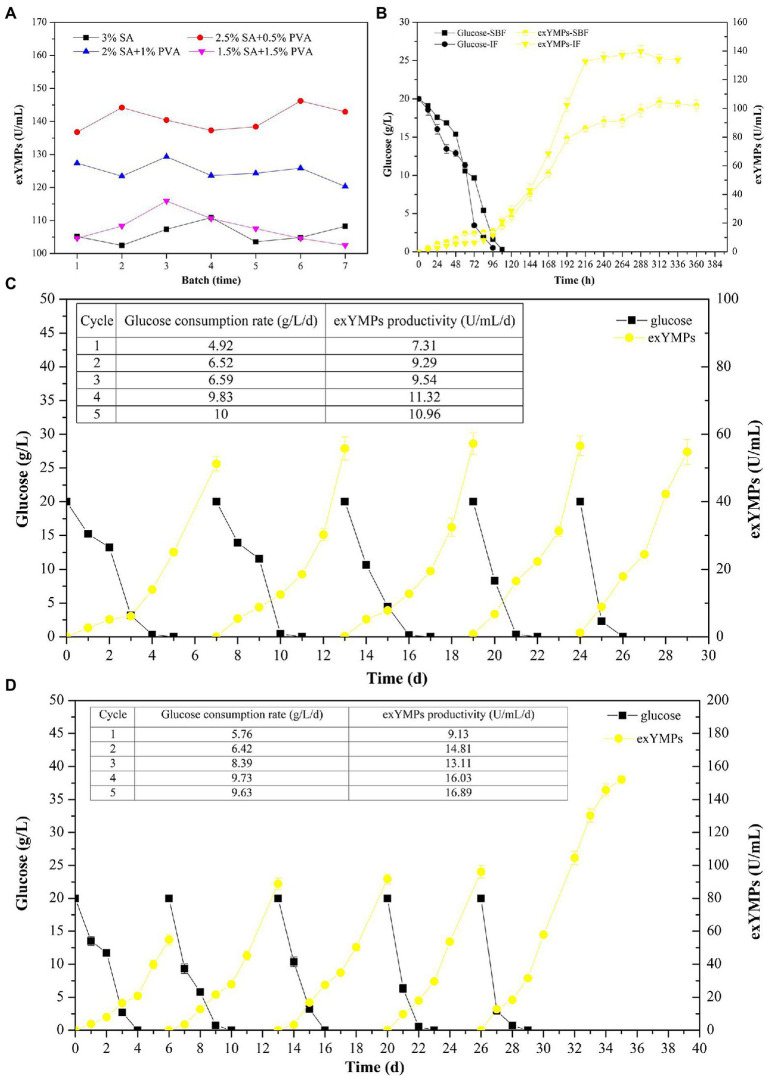
The effect of different ratios of sodium alginate and polyvinyl alcohol solution on the production of extracellular yellow *monascus* pigments *via* repeated batch fermentation of *M. purpureus* H14 **(A)**; glucose consumption and the biosynthesis of extracellular yellow *monascus* pigments of *M. purpureus* H14 during conventional SBF and immobilized fermentation **(B)**; glucose consumption and the biosynthesis of extracellular yellow *monascus* pigments of *M. purpureus* H14 during repeated-batch fermentation without **(C)** and with **(D)** Triton X-100 addition.

It has been reported that the use of Ca-alginate to embed the mycelium of *M. purpureus* C322 resulted in high pigment production in RBF (Fenice et al., 2000). Our previous study investigated the effect of SA concentration on exMP production and immobilization efficiency and found that the optimal concentration of SA was 3% (wt/wt; Liu et al., 2019b). In this study, we further evaluated the effect of different SA and PVA ratios with a total concentration of 3% on exMP production and immobilization efficiency by immobilizing the spores of *M. purpureus* H14. As in our previous study, with a maximum of seven cycles, a large amount of batch fermentation could not be achieved in RBF (Figure 4A). In addition, although their immobilization efficiencies were good, exYMP production was different with different SA and PVA ratios in RBF with each cycle for 10 days. In descending order of exYMP production, they can be ranked as 2.5% SA and 0.5% PVA, 2% SA and 1% PVA, 1.5% SA and 1.5% PVA, and 3% SA, with mean values of 140.90, 124.90, 107.72, and 106.06 U/ml, respectively. The immobilization efficiency of *M. purpureus* H14 was desirable when the concentration of SA in the immobilized solution was less than 1.5%; it was flocculent and irregular, but not regularly spherical. Concentrations of 2.5% SA and 0.5% PVA in the immobilized solution were selected for immobilization of *M. purpureus* H14 spores in IF.

As shown in Figure 4B, the glucose consumption rate and exYMP biosynthesis rate were increased in IF compared to those in conventional SBF. The residual glucose was consumed at approximately 96 and 108 h, with glucose consumption rates of 0.21 g/L/h (5.04 g/L/day, increased by 16.67%) and 0.18 g/L/h (4.32 g/L/day) in IF and SBF, respectively. The biosynthesis rate of exYMPs using *M. purpureus* H14 was evenly matched in the mid-early period of fermentation (before 144 h) in SBF and IF; subsequently, the biosynthesis rate significantly increased in IF compared to that in SBF, with a maximum exYMP production of 139.75 U/ml at 288 h (a productivity of 11.64 U/ml/day) and 104.13 U/ml at 300 h (a productivity of 8.33 U/ml/day), respectively. In RBF without the addition of Triton X-100 for five cycles, the mean glucose consumption rate and exYMP productivity were 8.23 and 10.28 U/ml/day, respectively, not including the first batch (Figure 4C). However, these values were increased to 8.54 g/L/day and 15.21 U/ml/day, respectively, in RBF with Triton X-100 (Figure 4D). Similarly, Evans and Wang (1984) further increased the maximum *Monascus* pigment yield and production rate through immobilized cell fermentation with the addition of resin, illustrating that a novel RBF with some modifications could increase the yield and productivity of a target product. To evaluate the maximum exYMP production using this novel RBF, we extended the fermentation time to 10 days (the same as conventional SBF) in the last cycle. An exYMP production of 152.08 U/ml was obtained, and the exYMP productivity was almost constant (16.89 U/ml/day). The exYMP yield and productivity increased by 570.55 and 570.04%, respectively, compared with SBF after RSM and RBF (22.68 U/ml and 2.27 U/ml/day).

In addition, we compared the yield and productivity of YMPs produced by *Monascus* strains *via* SBF. As shown in Table 4, various biotechnologies were carried out to increase the YMPs production, including addition of exogenous compounds [such as ethanol, sodium starch octenyl succinate (OSA-SNa), and microparticle], pH and sharking speed regulation, temperature control, and response surface methodology, changing oxidation–reduction potential (high glucose stress, addition of H_2_O_2_ and dithiothreitol). The highest exYMPs production was 209 U/ml in submerged fermentation by *M. ruber* CGMCC 10910 after optimizing the amount of H_2_O_2_ added and the timing of the addition, with 17.42 U/ml/day of productivity (Huang et al., 2021b), which was similar to the value of that obtained in this study. Besides, it showed that high production of YMPs (328.36 U/ml) was obtained by *M. ruber* CGMCC 10910 under condition of high glucose stress, but the productivity of exYMPs was low (5.22 U/ml/day; Wang et al., 2017b). Although most YMPs productions were much higher than that in this present study, the productivity of exYMPs generated *via* RBF was advantageous and predominant.

**Table 4:** Comparison of the production and productivity of yellow *monascus* pigments by *Monascus* strains in submerged fermentation.

Strain	Biotechnology	exYMPs (U/ml)	T-YMPs (U/ml)	Productivity (U/ml/day)	Reference
*M. ruber* CGMCC 10910	Temperature control	190	–	23.75 (exMPs)	Huang et al., 2017a
*M. ruber* CGMCC 10910	Controlling oxidoreduction potential	209	–	17.42 (exMPs)	Huang et al., 2017b
*M. ruber* CGMCC 10910	High glucose stress	114.80	328.36	5.22 (exMPs)	Wang et al., 2017b
*M. ruber* CGMCC 10910	Addition of OSA-SNa	131.50	220	16.44 (exMPs)	Huang et al., 2021b
*M. anka* mutant MYM	Response surface methodology	–	87.24	12.46 (T-YMPs)	Zhou et al., 2009
*M. purpureus* sjs-6	Adjusting pH and shaking speed	–	401	57.28 (T-YMPs)	Lv et al., 2017
*M. purpureus* ZH106-E	Addition of ethanol	–	394	56.29 (T-YMPs)	Qian et al., 2021
*M. purpureus* CH01	Potato pomace as carbon source	–	19.70	2.81 (T-YMPs)	Chen et al., 2021
*M. purpureus* ZH106-M	Addition of microparticles		554.20	79.17 (T-YMPs)	Huang et al., 2021a
*M. purpureus* H14	Immobilized fermentation	152.08	–	16.89 (exMPs)	In this study

### Physicochemical Property of Produced Yellow *Monascus* Pigments

**Figure 5:**
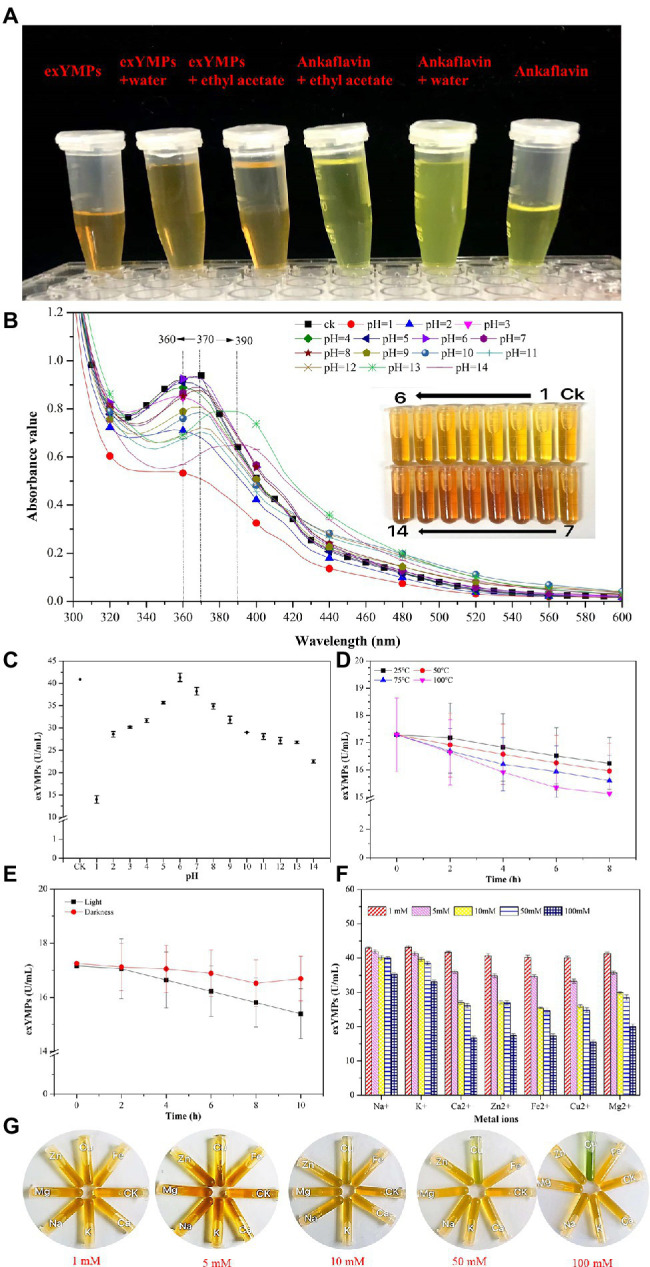
The polar analysis of extracellular yellow *monascus* pigments produced by the mutant strain *M. purpureus* H14 **(A)**; spectral scan results **(B)** and the value **(C)** of extracellular yellow *monascus* pigments in a pH range of 1–14; the value stability of extracellular yellow *monascus* pigments produced by the mutant strain *M. purpureus* H14 subject to heat **(D)**, light **(E)**, metal ions **(F)**; the tone of extracellular yellow *monascus* pigments in response to various metal ions with different concentrations **(G)**.

**Figure 6:**
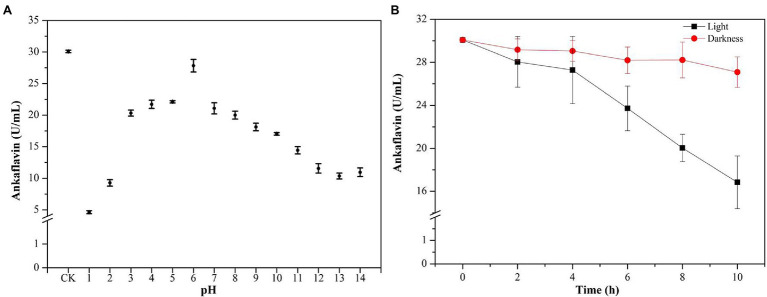
The value stability of ankaflavin subject to pH **(A)** and light **(B)**.

Feng et al. (2012) reviewed the categories, structures, and properties (including solubility, stability, and safety) of MPs in the journal of Applied Microbiology Biotechnology. YMPs (absorption at 420 ± 10 nm), OMPs (absorption at 470 ± 10 nm), and RMPs (absorption at 510 ± 10 nm) are traditionally accumulated in the mycelium during SBF (Kim and Ku, 2018). The four identified MPs, N-glutarylmonascorubramine, N-glutarylrubropunctamine, N-glucoslmonascorubramine, and N-glucosylrubropunctamine are hydrophilic MPs (exMPs) with a red tone (Chen et al., 2015). In this study, a large number of YMPs produced by the mutant strain *M. purpureus* H14 in SBF were secreted out of the cell; we speculated that the exYMPs were hydrophilic or water-soluble. From the polarity test (Figure 5A), we found that the exYMPs were soluble in pure water, but were not dissolved in the hydrophobic phase of ethyl acetate. However, ankaflavin, an alcohol-soluble standard compound, is easily dissolved in the hydrophobic phase. These observations verify that the WSYMP hypothesis was correct. Figure 5B shows that the yellow tone of the produced WSYMPs is stable in a pH range of 1–14. However, the maximum absorption peaks are different: 360 nm for pH 1–5, 370 nm for pH 6–12, and 390 nm for pH 13–14. The maximum absorption peaks for WSYMPs were different from those of YMPs. However, the WSYMP values changed with the addition of 1 M HCl or NaOH, decreasing to 13.98 U/ml (65.84%) and 22.52 U/ml (44.97%) from 40.92 U/ml at pH 1 and pH 14, respectively (Figure 5C). The produced WSYMPs have high stability in neutral solution environments, decreasing in alkaline and acidic environments. Similarly, the color value degraded at different pH values; degradation was more significant from pH 4–8 (Carvalho et al., 2005). Although the color tone was changeless, Figure 6A shows that ankaflavin has relatively high stability in a pH range of 3–8, the value dramatically decreased when pH lower than 2 (decreased by more than 79.15%) and more than 9 (decreased by more than 43.35%). The results stated that the generated WSYMPs by mutant strain *M. purpureus* H14 have higher stability in response to pH than ankaflavin. Besides, in Figure 5D, the WSYMPs are almost stable with heat treatment (25–90°C) for 8 h, exhibiting a slight reduction in yellow tone. It has been reported that MPs are generally stable with heat and a wide range of pH values, but are less stable with light (Wang et al., 2017a). However, traditionally the focus is tone stability, without considering the value. Water-soluble RMPs were generated by chemical modification using aminoacetic acid and glutamic acid and exhibited heat discoloration when dissolved in a buffer at pH 3, 7 and 9.2 (Wong and Koeler, 1983). A type of RMP produced by *M. purpureus* using corn bran in SBF produced thermal (sterilization and 40°C–80°C) and pH (4–7) stability for tone and value (Almeida et al., 2021). In this study, the WSYMP value was almost constant in the dark for 10 h, exhibiting a downward trend over time (0–10 h) in light, but remaining nearly constant with an initial value of 17.16 U/ml until 2 h (Figure 5E). Similarly, the color value of ankaflavin sharply decreased to 16.84 U/ml for ten hours from 30.08 U/ml in light, but almost constant in the dark (Figure 6B). However, the stability of WSYMPs is relatively higher than that of ankaflavin when they are irradiated within 2 h. Figure 5F shows that the WSYMPs were stable in Na^+^ and K^+^ solutions, with low concentrations (<5 mM) of Ca^2+^, Zn^2+^, Fe^2+^, Cu^2+^, and Mg^2+^. However, when the concentration reached 10 mM, the pigment value decreased by 32.60, 32.16, 36.35, 35.03, and 25.30%, and 52.62, 50.63, 50.90, 55.97, and 43.43%, respectively, at a concentration of 100 mM. The pigment tone changed to green when the Cu^2+^ concentration exceeded 50 mM; the YMPs solution with the addition of other metal ions maintained a yellow tone (Figure 5G). Traditionally, MPs are stable with a small quantity of Na^+^, K^+^, Mg^2+^, Ca^2+^, Zn^2+^, Al^3+^, and Cu^2+^, but less stable in the presence of Fe^2+^ and Fe^3+^ (Feng et al., 2012; Wang et al., 2017a). These findings illustrated that the WSYMPs showed good stability of tone to environmental factors, including pH, heat, light, and metal ions, in this present study. However, the pigment value was unstable to pH, light, and high concentrations of Ca^2+^, Zn^2+^, Fe^2+^, Cu^2+^, and Mg^2+^.

### Characterization of Produced Yellow *Monascus* Pigments

**Figure 7:**
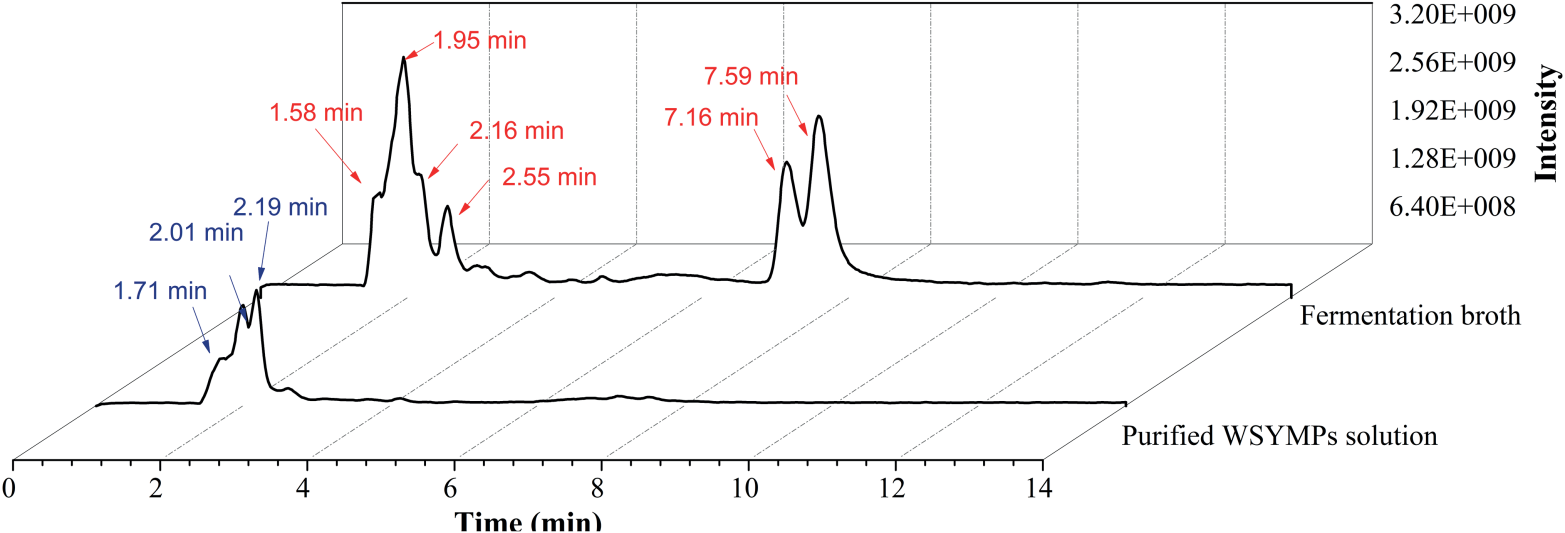
Ultra-high-performance liquid chromatography analysis of fermentation broth and WSYMP solution.

**Figure 8:**
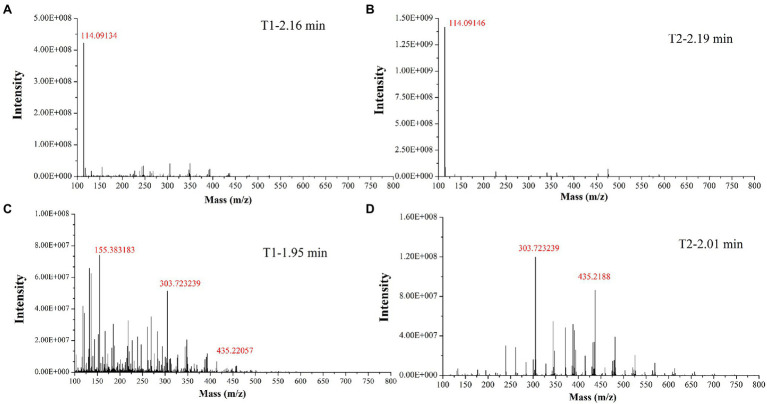
Identification of yellow *monascus* pigment from WSYMP solution and fermentation broth by UHPLC-ESI-MS.

**Figure 9:**
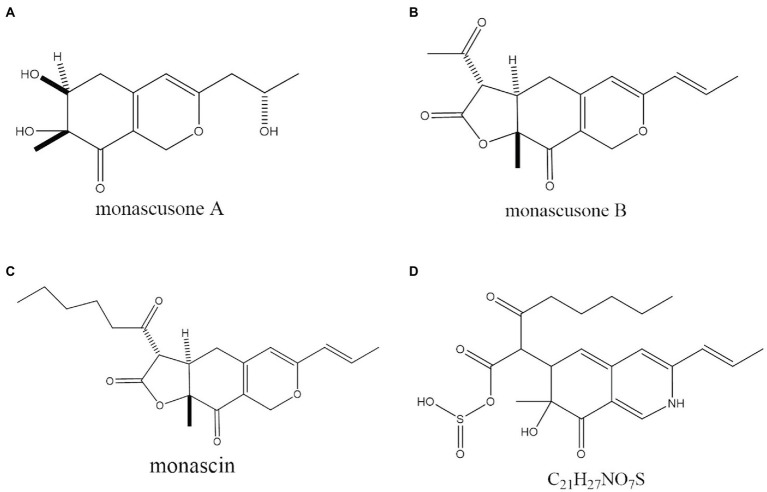
The chemical structure of monascusone A **(A)**, monascusone B **(B)**, and monascin **(C)**, and C_21_H_27_NO_7_S **(D)**.

In the past decade, 12 YMP compounds have been investigated (Feng et al., 2012). In this study, we found that the HPLC peak time of produced WSYMPs different to that of ankaflavin and monascin in our laboratory. Thus, we further determined the compound structures of the YMPs through HPLC–MS. From Figure 7, a total of five peaks were appeared in the raw fermentation broth (the first tested sample, T1), and the retention time (*t*_R_) was 1.58, 1.95, 2.16, 2.55, 7.16, and 7.59 min, respectively. However, the peaks with *t*_R_ of 2.55, 7.16, and 7.59 disappeared in the WSYMPs solution (the second tested sample, T2), and the *t*_R_ of residual three peaks occurred rearward shift within the range of recognition, which changed to 1.71, 2.01, and 2.19 min, respectively. Besides, the compounds with *t*_R_ of 7.16 and 7.59 min both showed pseudo-molecular [M + H]^+^ ions at m/z 301.14 and 301.14, with the fragment ions in UPLC-ESI-MS^2^ at m/z 149.02343 and 163.03858. The fragment ions of the compound with *t*_R_ of 2.55 min in UHPLC-ESI-MS^2^ at m/z 362.32822, 300.28851, and 265.26337. This phenomenon illustrated that some compounds or impurities (not WSYMPs) were cleared away *via* the adsorption–separation system with the macroporous adsorption resin LX300C. Furtherly, we analyzed the peaks with t_R_ of 2.01 min (or 1.95 min) and 2.19 min (or 2.16 min) by UHPLC-ESI-MS. The peaks of T1-2.16 and T2-2.19 min both showed pseudo-molecular [M + H]^+^ ions at m/z 114.09134 (Figure 8). Although a pseudo-molecular [M + H] ^+^ ion with m/z 155.383183 in T1 − 1.95 min, it disappeared after purification treatment (in T2 2.01 min). In addition, the peak of T1-1.95 min mainly showed pseudo-molecular [M + H] ^+^ ions at m/z 303.723239, 435.33057, and that of T2-2.01 min at m/z 303.723239 and 435.2188. Furtherly, the components of exYMPs solution were separated *via* dynamic desorption of fully saturated absorbent with glass absorbent columns of 200 mm length and 10 mm diameter. The sample with the darkest yellow color tone was analyzed by Nanjing Sheng Ming Yuan Health Technology Co. Ltd., and the result showed that the molecular weight of component was m/z at 440.1150 of [M + H]^+^ (Supplementary Figure S1—HPLC and Supplementary Figure S2—MS). Compared with the information in studies, we found that the molecular, monascusone B (C_17_H_18_O_5_), has a weight of m/z 302 [observed m/z at 303.1235 of (M + H)^+^] and UV *λ*_max_ value at 375 nm (Jongrungruangchok et al., 2004); compound **7** (C_21_H_27_NO_7_S, a kind of WSYMPs) observed m/z at 435.7 of [M + H]^+^ (Yang et al., 2018). The results indicated that most possibly only two yellow *Monascus* pigment components were produced by mutant strain *M. purpureus* H14 in submerged fermentation, and the exYMP was identified as monascusone B and C_21_H_27_NO_7_S (WSYMP; the structures are shown in Figure 9).

Besides, Jongrungruangchok et al. (2004) have reported that monascusone A (a precursor substance of monascusone B) produced by *M. kaoliang* KB20M10.2 showed no cytotoxicity against breast cancer and human epidermoid carcinoma of cavity cell lines. Monascusone B possesses the same stereochemistry as that of monascin, which exhibited no cytotoxicity against Hep G2 (human cancer cell lines) cells (Su et al., 2005). In addition, the IC50 of ankaflavin on human cancer cell lines is 15 μg/ml, but ankaflavin has no significant cytotoxicity against on normal diploid fibroblast cell lines (Su et al., 2005). These results indicated that the constructed fermentation system (including dual mutagenesis, immobilized fermentation, and extractive fermentation) is beneficial for the production of exYMP in submerged fermentation by *M. purpureus*.

## Conclusion

Yellow *Monascus* pigments are excellent colorants, are safe, and have several physiological functions. In this study, a mutant strain, *M. purpureus* H14, was generated using dual mutagenesis of atmospheric and room-temperature plasma and HIBI. The physicochemical property of the produced YMP was water-soluble, and two yellow monascus pigment components were identified by UHPLC-ESI-MS. It exhibited good tone stability when subjected to environmental factors including pH, heat, light, and metal ions, but the pigment value was unstable with pH, light, and high concentrations of Ca^2+^, Zn^2+^, Fe^2+^, Cu^2+^, and Mg^2+^. Application of IF and EF significantly improved the exYMP yield and productivity.

## Data Availability Statement

The data analyzed in this study is subject to the following licenses/restrictions. The data presented in this study are available on request from the corresponding author. Requests to access these datasets should be directed to JL, liujundandy@csuft.edu.cn.

## Author Contributions

JL and QL designed experiments. JB, ZG, and HZ performed experiments. JB wrote the manuscript. JL revised the manuscript. MS collected important background information. FY analyzed the RSM results. JL and BZ analyzed the data. DL and XZ performed heavy ion-beam irradiation. CT analyzed the HPLC-MS data. All authors contributed to the article and approved the submitted version.

## Funding

This work was supported by the National Natural Science Foundation of China (no. 32101906), Hunan Provincial Natural Science Foundation (no. 2021JJ31146), Open Project Program of the Hunan Provincial Key Laboratory of Food Safety Monitoring and Early Waring (no. 2021KFJJ02), and Education Department of Scientific Research Project of Hunan Province (no. 20B619), and Education Department of Postgraduate Research and Innovation Project of Hunan Province (no. CX20210863).

## Conflict of Interest

CT was employed by Nanjing Sheng Ming Yuan Health Technology Co. Ltd. and Jiangsu Institute of Industrial Biotechnology JITRI Co. Ltd.

The remaining authors declare that the research was conducted in the absence of any commercial or financial relationships that could be construed as a potential conflict of interest.

## Publisher’s Note

All claims expressed in this article are solely those of the authors and do not necessarily represent those of their affiliated organizations, or those of the publisher, the editors and the reviewers. Any product that may be evaluated in this article, or claim that may be made by its manufacturer, is not guaranteed or endorsed by the publisher.

## References

[ref1] Almeida A. B. D. Santos N. H. Lima T. M. D. Santana R. V. de Oliveira Filho J. G. Peres D. S.. (2021). Pigment bioproduction by *Monascus purpureus* using corn bran, a byproduct of the corn industry. Biocatal. Agric. Biotechnol. 32:101931. doi: 10.1016/j.bcab.2021.101931

[ref2] Carvalho J. C. D. Oishi B. O. Pandey A. Soccol C. R. (2005). Biopigments from *Monascus*: strains selection, citrinin production and color stability. Braz. Arch. Biol. Technol. 48, 885–894. doi: 10.1590/S1516-89132005000800004

[ref3] Chen G. Bei Q. Huang T. Wu Z. (2017a). Tracking of pigment accumulation and secretion in extractive fermentation of *Monascus anka* GIM 3.592. Microb. Cell Factories 16:172. doi: 10.1186/s12934-017-0786-6, PMID: 28978326 PMC5628469

[ref4] Chen X. J. Chen M. M. Wu X. F. Li X. J. (2021). Cost-effective process for the production of *Monascus* pigments using potato pomace as carbon source by fed-batch submerged fermentation. Food Sci. Nutr. 9, 5415–5427. doi: 10.1002/fsn3.2496, PMID: 34646512 PMC8497832

[ref5] Chen W. He Y. Zhou Y. Shao Y. Feng Y. Li M.. (2015). Edible filamentous Fungi from the species Monascus: early traditional fermentations, modern molecular biology, and future genomics. Compr. Rev. Food Sci. Food Saf. 14, 555–567. doi: 10.1111/1541-4337.12145

[ref6] Chen G. Huang T. Bei Q. Tian X. Wu Z. (2017b). Correlation of pigment production with mycelium morphology in extractive fermentation of *Monascus anka* GIM 3.592. Process Biochem. 58, 42–50. doi: 10.1016/j.procbio.2017.04.012

[ref7] Chen M. H. Johns M. R. (1993). Effect of pH and nitrogen source on pigment production by *Monascus purpureus*. Appl. Microbiol. Biotechnol. 40, 132–138. doi: 10.1007/BF00170441

[ref8] Chen M. H. Johns M. R. (1994). Effect of carbon source on ethanol and pigment production by *Monascus purpureus*. Enzym. Microb. Technol. 16, 584–590. doi: 10.1016/0141-0229(94)90123-6

[ref9] Chen L. Tang H. Du Y. Dai Z. Wang T. Wu L.. (2018). Induction of reproductive cell death in *Caenorhabditis elegans* across entire linear-energy-transfer range of carbon-ion irradiation. DNA Repair 63, 39–46. doi: 10.1016/j.dnarep.2018.01.009, PMID: 29414052

[ref10] Dong T. T. Gong J. S. Gu B. C. Zhang Q. Li H. Lu Z. M.. (2017). Significantly enhanced substrate tolerance of *Pseudomonas putida* nitrilase via atmospheric and room temperature plasma and cell immobilization. Bioresour. Technol. 244, 1104–1110. doi: 10.1016/j.biortech.2017.08.039, PMID: 28873512

[ref11] Evans P. J. Wang H. Y. (1984). Pigment production from immobilized *Monascus* sp. utilizing polymeric resin adsorption. Appl. Environ. Microbiol. 47, 1323–1326. doi: 10.1128/aem.47.6.1323-1326.1984, PMID: 16346570 PMC240234

[ref12] Feng Y. Shao Y. Chen F. (2012). Monascus pigments. Appl. Microbiol. Biotechnol. 96, 1421–1440. doi: 10.1007/s00253-012-4504-3 23104643

[ref13] Fenice M. Federici F. Selbmann L. Petruccioli M. (2000). Repeated-batch production of pigments by immobilised *Monascus purpureus*. J. Biotechnol. 80, 271–276. doi: 10.1016/S0168-1656(00)00271-6, PMID: 10949317

[ref14] Gao Y. Zhang M. Zhou X. Guo X. Lu D. (2020). Effects of carbon ion beam irradiation on Butanol tolerance and production of *Clostridium acetobutylicum*. Front. Microbiol. 11:602774. doi: 10.3389/fmicb.2020.602774, PMID: 33391222 PMC7775398

[ref15] Gong C. Wu Z. (2016). Production and biological activities of yellow pigments from *Monascus* fungi. World J. Microbiol. Biotechnol. 32:136. doi: 10.1007/s11274-016-2082-8, PMID: 27357404

[ref16] Guo X. Zhang M. Gao Y. Lu D. Zhou L. (2020). Repair characteristics and time-dependent effects in response to heavy-ion beam irradiation in *Saccharomyces cerevisiae*: a comparison with X-ray irradiation. Appl. Microbiol. Biotechnol. 104, 4043–4057. doi: 10.1007/s00253-020-10464-8, PMID: 32144474

[ref17] Hu Z. Zhang X. Wu Z. Qi H. Wang Z. (2012). Export of intracellular *Monascus* pigments by two-stage microbial fermentation in nonionic surfactant micelle aqueous solution. J. Biotechnol. 162, 202–209. doi: 10.1016/j.jbiotec.2012.10.004, PMID: 23079078

[ref18] Huang J. Guan H. W. Huang Y. Y. Lai K. S. Chen H. Y. Xue H.. (2021a). Evaluating the effects of microparticle addition on mycelial morphology, natural yellow pigments productivity, and key genes regulation in submerged fermentation of *Monascus purpureus*. Biotechnol. Bioeng. 118, 2503–2513. doi: 10.1002/bit.27762, PMID: 33755193

[ref19] Huang T. Tan H. Chen G. Wang L. Wu Z. (2017a). Rising temperature stimulates the biosynthesis of water-soluble fluorescent yellow pigments and gene expression in *Monascus ruber* CGMCC10910. AMB Express 7:134. doi: 10.1186/s13568-017-0441-y, PMID: 28651383 PMC5483225

[ref20] Huang T. Tan H. Lu F. Chen G. Wu Z. (2017b). Changing oxidoreduction potential to improve water-soluble yellow pigment production with *Monascus ruber* CGMCC 10910. Microb. Cell Factories 16:208. doi: 10.1186/s12934-017-0828-0, PMID: 29162105 PMC5697053

[ref21] Huang T. Wang M. Shi K. Chen G. Tian X. Wu Z. (2017c). Metabolism and secretion of yellow pigment under high glucose stress with *Monascus ruber*. AMB Express 7:79. doi: 10.1186/s13568-017-0382-5, PMID: 28401504 PMC5388664

[ref22] Huang Z. F. Yang S. Z. Liu H. Q. Tian X. F. Wu Z. Q. (2021b). Sodium starch octenyl succinate facilitated the production of water-soluble yellow pigments in *Monascus ruber* fermentation. Appl. Microbiol. Biotechnol. 105, 6691–6706. doi: 10.1007/s00253-021-11512-7, PMID: 34463799

[ref23] Jongrungruangchok S. Kittakoop P. Yongsmith B. Bavovada R. Tanasupawat S. Lartpornmatulee N.. (2004). Azaphilone pigments from a yellow mutant of the fungus *Monascus* kaoliang. Phytochemistry 65, 2569–2575. doi: 10.1016/j.phytochem.2004.08.032, PMID: 15451319

[ref24] Kim D. Ku S. (2018). Beneficial effects of *Monascus* sp. KCCM 10093 pigments and derivatives: a mini review. Molecules 23:98. doi: 10.3390/molecules23010098, PMID: 29301350 PMC6017178

[ref25] Kodym A. Afza R. (2003). Physical and Chemical Mutagenesis. Methods in Molecular Biology 236, 189–204. 14501066 10.1385/1-59259-413-1:189

[ref26] Lai J. R. Hsu Y. W. Pan T. M. Lee C. L. (2021). Monascin and Ankaflavin of *Monascus purpureus* prevent alcoholic liver disease through regulating AMPK-mediated lipid metabolism and enhancing Both anti-inflammatory and anti-oxidative systems. Molecules 26:6301. doi: 10.3390/molecules26206301, PMID: 34684882 PMC8538843

[ref27] Liu J. Chai X. Guo T. Wu J. Yang P. Luo Y.. (2019a). Disruption of the Ergosterol biosynthetic pathway results in increased membrane permeability, causing overproduction and secretion of extracellular *Monascus* pigments in submerged fermentation. J. Agric. Food Chem. 67, 13673–13683. doi: 10.1021/acs.jafc.9b05872, PMID: 31617717

[ref28] Liu J. Guo T. Luo Y. Chai X. Wu J. Zhao W.. (2019b). Enhancement of *Monascus* pigment productivity via a simultaneous fermentation process and separation system using immobilized-cell fermentation. Bioresour. Technol. 272, 552–560. doi: 10.1016/j.biortech.2018.10.072, PMID: 30396112

[ref29] Liu J. Luo Y. Guo T. Tang C. Chai X. Zhao W.. (2020b). Cost-effective pigment production by *Monascus purpureus* using rice straw hydrolysate as substrate in submerged fermentation. J. Biosci. Bioeng. 129, 229–236. doi: 10.1016/j.jbiosc.2019.08.007, PMID: 31500988

[ref30] Liu L. Wu S. Wang W. Zhang X. Wang Z. (2020a). Sulfonation of *Monascus* pigments to produce water-soluble yellow pigments. Dyes Pigments 173:107965. doi: 10.1016/j.dyepig.2019.107965

[ref31] Lu P. Wu A. Zhang S. Bai J. Guo T. Lin Q.. (2021). Triton X−100 supplementation regulates growth and secondary metabolite biosynthesis during in-depth extractive fermentation of *Monascus purpureus*. J. Biotechnol. 341, 137–145. doi: 10.1016/j.jbiotec.2021.09.018, PMID: 34601020

[ref32] Lv J. Zhang B. B. Liu X. D. Zhang C. Chen L. Xu G. R.. (2017). Enhanced production of natural yellow pigments from Monascus purpureus by liquid culture: the relationship between fermentation conditions and mycelial morphology. J. Biosic. Bioeng. 124, 452–458. doi: 10.1016/j.jbiosc.2017.05.010, PMID: 28625612

[ref33] Qian G. F. Huang J. Farhadi A. Zhang B. B. (2021). Ethanol addition elevates cell respiratory activity and causes overproduction of natural yellow pigments in submerged fermentation of *Monascus purpureus*. LWT Food Sci. Technol. 139:110534. doi: 10.1016/j.lwt.2020.110534

[ref34] Shi K. Song D. Chen G. Pistolozzi M. Wu Z. Quan L. (2015). Controlling composition and color characteristics of *Monascus* pigments by pH and nitrogen sources in submerged fermentation. J. Biosci. Bioeng. 120, 145–154. doi: 10.1016/j.jbiosc.2015.01.001, PMID: 25648278

[ref35] Srivastav P. Yadav V. K. Govindasamy S. Chandrasekaran M. (2015). Red pigment production by *Monascus purpureus* using sweet potato-based medium in submerged fermentation. Forum Nutr. 14, 159–167. doi: 10.1007/s13749-015-0032-y

[ref36] Su N. W. Lin Y. L. Lee M. H. Ho C. Y. (2005). Ankaflavin from Monascusfermented red rice exhibits selective cytotoxic effect and induces cell death on Hep G2 cells. J. Agric. Food Chem. 53, 1949–1954. doi: 10.1021/jf048310e, PMID: 15769119

[ref37] Sun J. L. Zou X. Liu A. Y. Xiao T. F. (2011). Elevated yield of Monacolin K in *Monascus purpureus* by fungal elicitor and mutagenesis of UV and LiCl. Biol. Res. 44, 377–382. doi: 10.4067/S0716-97602011000400010, PMID: 22446602

[ref38] Wang C. Chen D. Qi J. (2017a). “Biochemistry and molecular mechanisms of Monascus pigments” in Bio-pigmentation and Biotechnological Implementations, 173–191.

[ref39] Wang M. Huang T. Chen G. Wu Z. (2017b). Production of water-soluble yellow pigments via high glucose stress fermentation of *Monascus ruber* CGMCC 10910. Appl. Microbiol. Biotechnol. 101, 3121–3130. doi: 10.1007/s00253-017-8106-y, PMID: 28091787

[ref40] Wong H. C. Koeler P. E. (1983). Production of red water-soluble *Monascus* pigments. J. Food Sci. 48, 1200–1203. doi: 10.1111/j.1365-2621.1983.tb09191.x

[ref41] Wu L. Zhou K. Chen F. Chen G. Yu Y. Lv X.. (2021). Comparative study on the antioxidant activity of *Monascus* yellow pigments From two different types of Hongqu—functional Qu and coloring Qu. Front. Microbiol. 12:715295. doi: 10.3389/fmicb.2021.715295, PMID: 34408740 PMC8365423

[ref42] Xiong X. Zhang X. Wu Z. Wang Z. (2015). Accumulation of yellow *Monascus* pigments by extractive fermentation in nonionic surfactant micelle aqueous solution. Appl. Microbiol. Biotechnol. 99, 1173–1180. doi: 10.1007/s00253-014-6227-0, PMID: 25417745

[ref43] Yang X. Dong Y. Liu G. Zhang C. Wang C. (2020). Effects of nonionic surfactants on pigment excretion and cell morphology in extractive fermentation of *Monascus* sp. NJ1. J. Sci. Food Agric. 100:1832. doi: 10.1002/jsfa.10171, PMID: 31825088

[ref44] Yang H. H. Li J. Wang Y. Gan C. J. (2018). Identification of water-soluble *Monascus* yellow pigments using HPLC-PAD-ELSD, high-resolution ESI-MS, and MS-MS. Food Chem. 245, 536–541. doi: 10.1016/j.foodchem.2017.10.121, PMID: 29287406

[ref45] Zhang X. Zhang X. F. Li H. P. Wang L. Y. Zhang C. Xing X. H.. (2014). Atmospheric and room temperature plasma (ARTP) as a new powerful mutagenesis tool. Appl. Microbiol. Biotechnol. 98, 5387–5396. doi: 10.1007/s00253-014-5755-y, PMID: 24769904

[ref46] Zhang S. Zhao W. Nkechi O. Lu P. Bai J. Lin Q.. (2022). Utilization of low-cost agricultural by-product rice husk for *Monascus* pigments production via submerged batch-fermentation. J. Sci. Food Agric. 102, 2454–2463. doi: 10.1002/jsfa.11585, PMID: 34642943

[ref47] Zhou W. Rui G. Guo W. Hong J. Lu L. Li N.. (2019). *Monascus* yellow, red and orange pigments from red yeast rice ameliorate lipid metabolic disorders and gut microbiota dysbiosis in Wistar rats fed on a high-fat diet. Food Funct. 10, 1073–1084. doi: 10.1039/C8FO02192A, PMID: 30720827

[ref48] Zhou B. Wang J. Pu Y. Zhu M. Liu S. Liang S. (2009). Optimization of culture medium for yellow pigments production with *Monascus* anka mutant using response surface methodology. Eur. Food Res. Technol. 228, 895–901. doi: 10.1007/s00217-008-1002-z

