# Supplementary figures and images for: Increased Water-Soluble Yellow Monascus Pigment Productivity via Dual Mutagenesis and Submerged Repeated-Batch Fermentation of Monascus purpureus
Source: Front Microbiol. 2022 Jun 9;13:914828. doi: 10.3389/fmicb.2022.914828 (PMC9218666; doi:10.3389/fmicb.2022.914828)

Y-2

05-May-2022

10:51:03

TL20220427044-1 Sm (Mn, 2x4)

ACQ-SQD2#NotSet

3: Diode Array

220

Range: 1.855e-1

Area

| Time  | Height | Area      | Area% |
|-------|--------|-----------|-------|
| 0.91  | 182182 | 14024.02  | 1.56  |
| 25.10 | 126719 | 884059.06 | 98.44 |

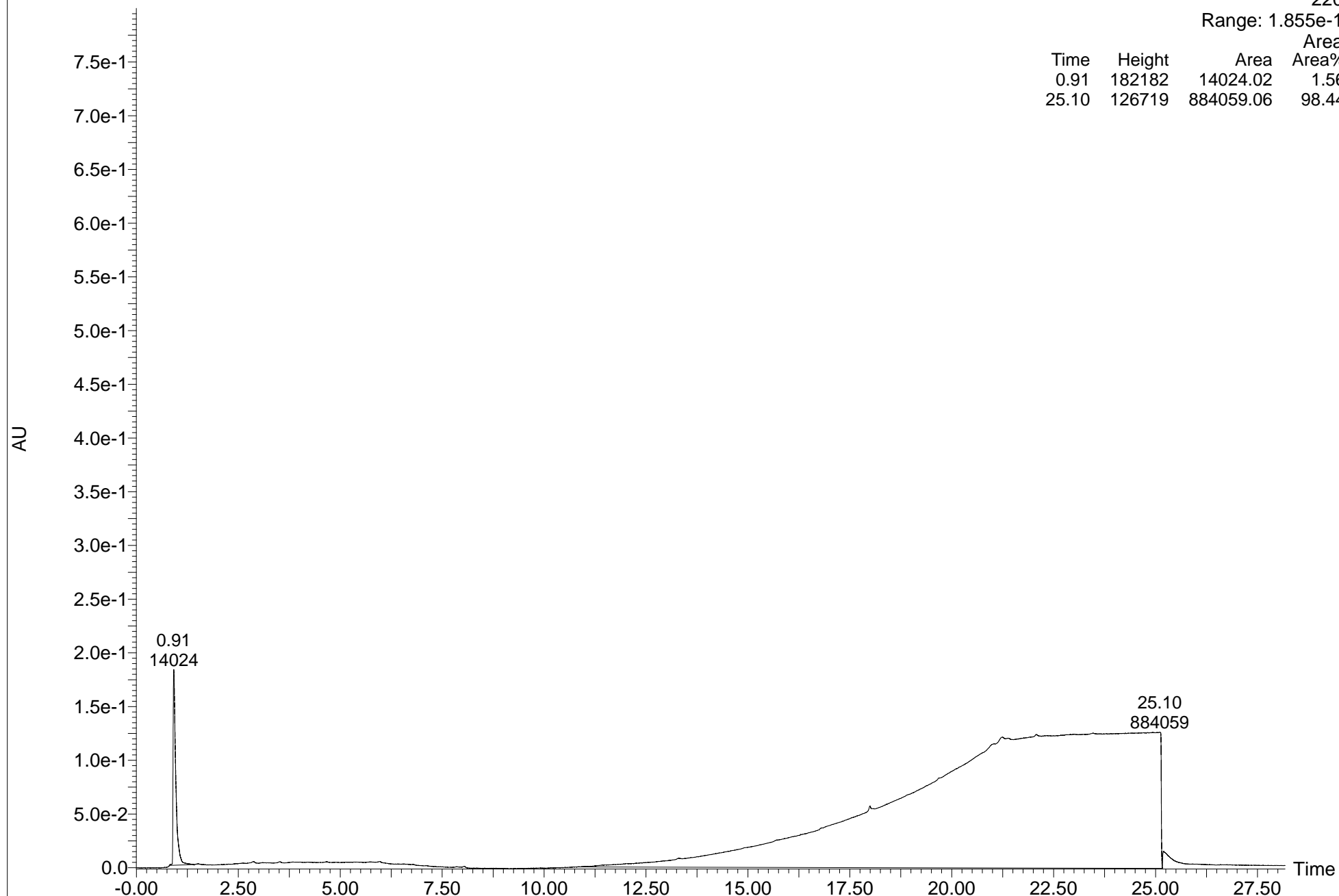

Supplement: Supplementary file 1 [file Data_Sheet_1.PDF]

Y-2

05-May-2022

10:51:03

TL20220427044-1 62 (1.067)

ACQ-SQD2#NotSet

1: Scan ES+  
5.46e6

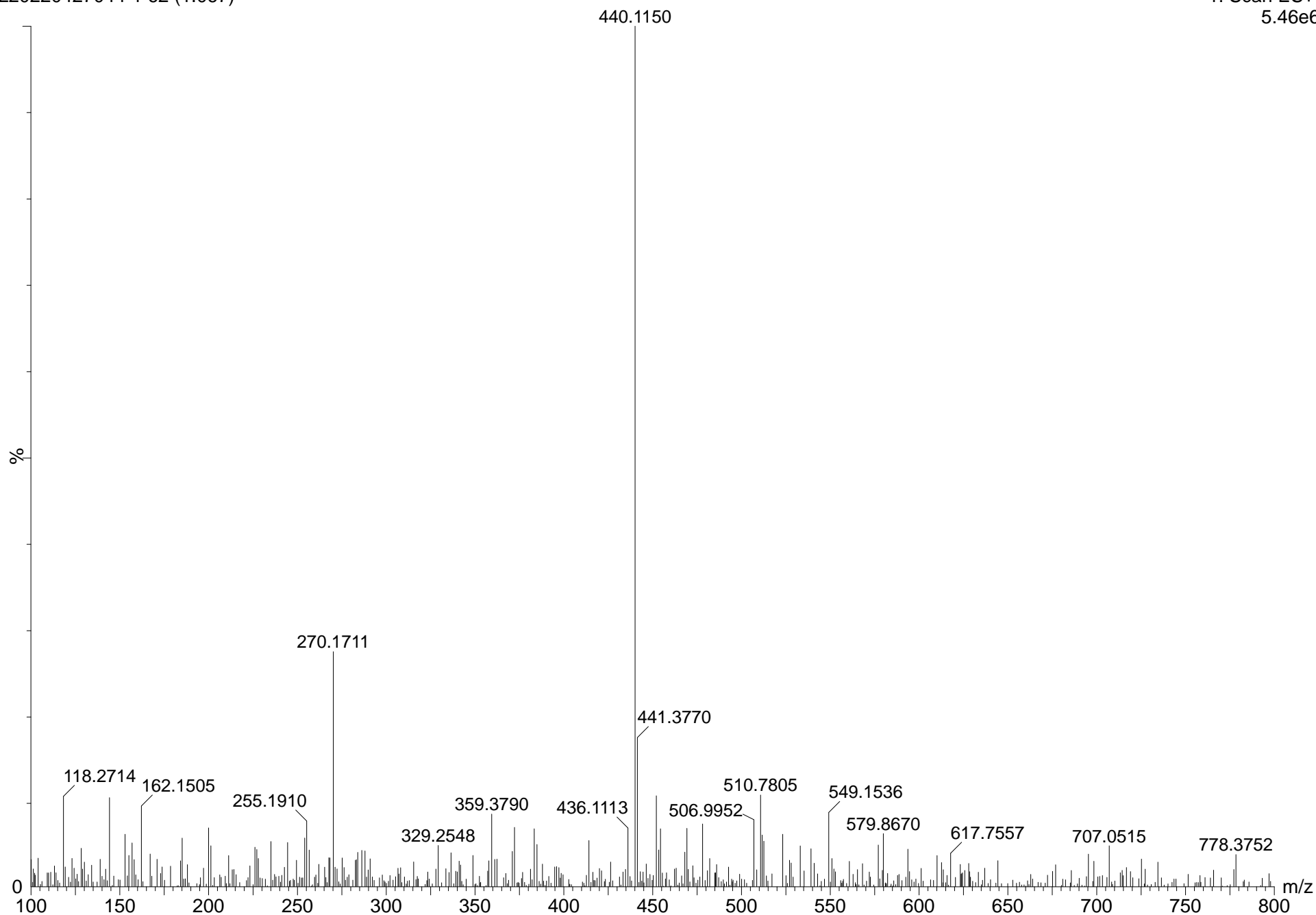

Supplement: Supplementary file 2 [file Data_Sheet_2.PDF]
